# Supplementary material for: Safe and Effective Delivery of mRNA Using Modified PEI-Based Lipopolymers
Source: Pharmaceutics. 2023 Jan 26;15(2):410. doi: 10.3390/pharmaceutics15020410 (PMC9967631; doi:10.3390/pharmaceutics15020410)
Supplement: Supplementary file 1 [file pharmaceutics-15-00410-s001.zip › pharmaceutics-2056530-supplementary.pdf]

Supporting information

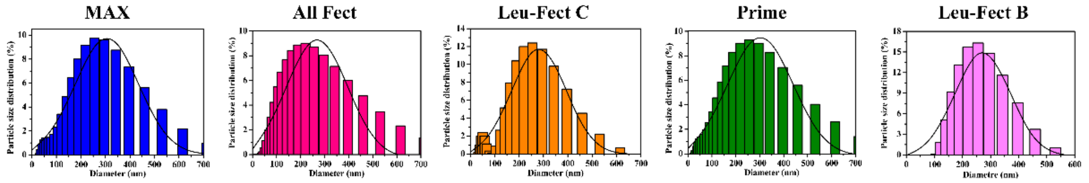

Figure S1. Particle size distribution of MAX, All Fect, Leu-Fect C, Prime, Leu-Fect B complexes as determined by DLS.

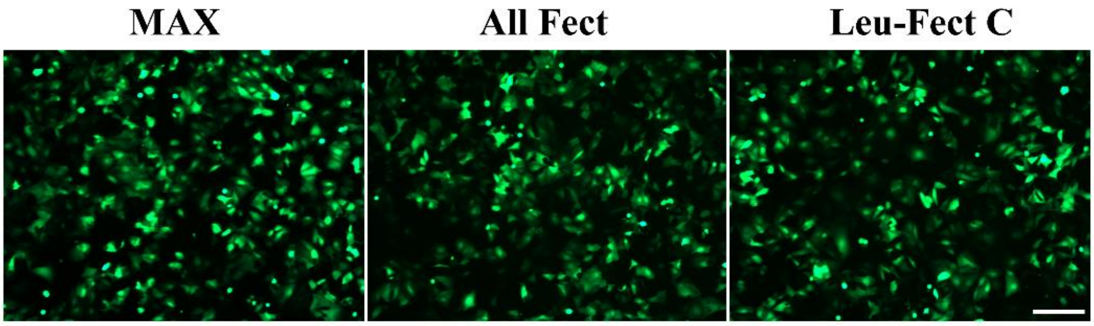

Figure S2. In vitro evaluation of GFP modRNA polymer vector transfection in MCF-7 cells. The scale bar is 200 μm.

Video S1

When polymer vectors delivered GFP modRNA into cardiomyocytes, it could still beat naturally and regularly.

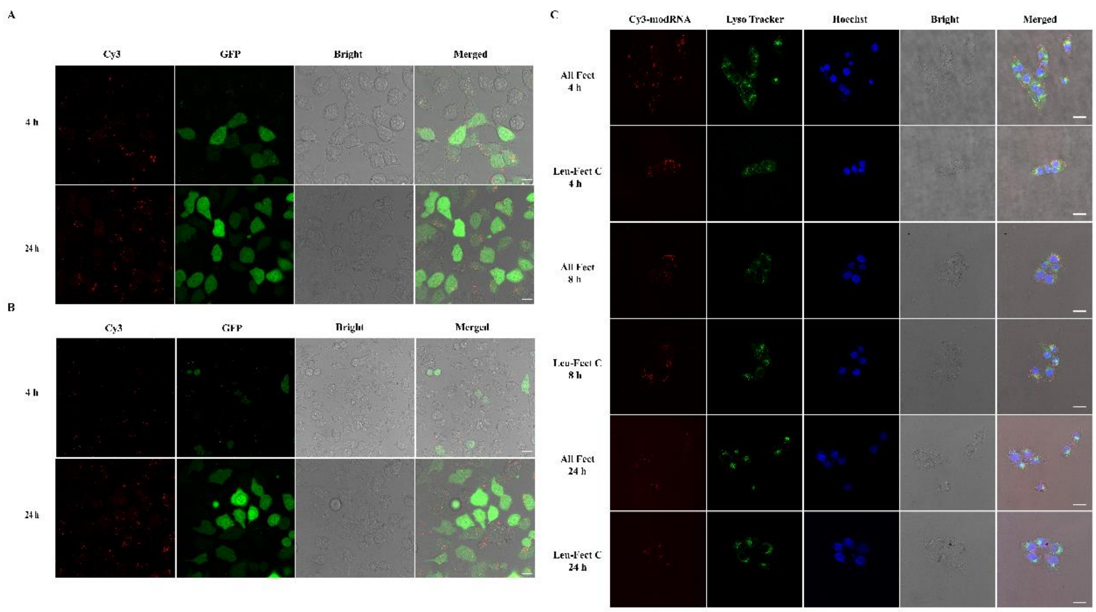

Figure S3. Cellular uptake and intracellular localization of modRNA-polymer complexes at different times in HeLa cells. (A) The ALL-Fect vector delivered Cy3-labeled GFP modRNA to HeLa cells and the localization of the complex in cells was observed under fluorescence microscopes at different times. (B) The Leu-Fect C vector delivered Cy3-labeled GFP modRNA to HeLa cells and the localization of the complex in cells was observed under fluorescence microscopes at different times. (C) Intracellular uptake images of Luciferase-modRNA polymer complexes in HeLa cells after different times, where cell nuclei were stained blue, lysosome was stained green, and modRNA was stained red. The scale bar is 25 μm.

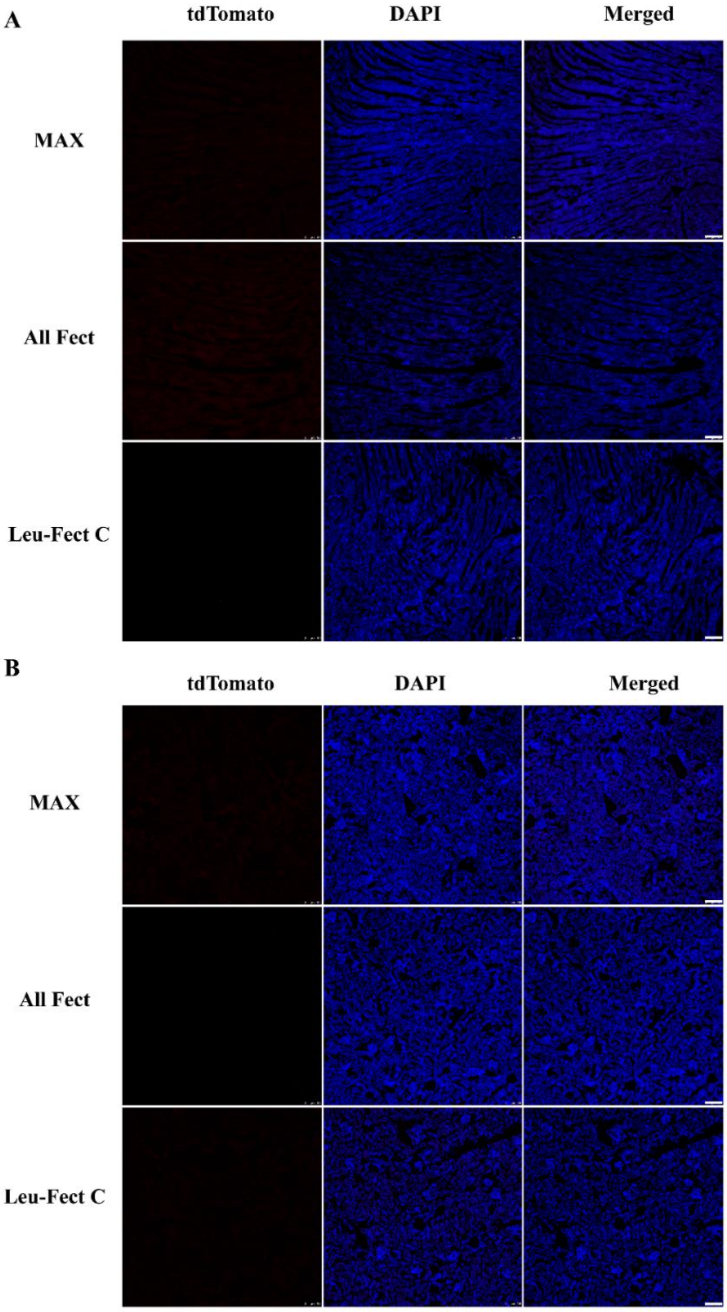

Figure S4. In vivo expression of Cre modRNA in the heart and kidney after tail vein injection. Three days after the injection of the modRNA polymer complexes into the tail vein, frozen sections of the heart and kidney were taken for staining to observe the effect of modRNA expression. (A) Immunofluorescence picture of in vivo transfection efficiency of Cre-modRNA in the heart. The scale bar is 100 μm. (B) Immunofluorescence picture of in vivo transfection efficiency of Cre modRNA in the kidney. The scale bar is 100 μm.
